# Micro‐RNAs: A safety net to protect hematopoietic stem cell self‐renewal

**DOI:** 10.1002/wrna.1693

**Published:** 2021-09-16

**Authors:** Laura Crisafulli, Francesca Ficara

**Affiliations:** ^1^ UOS Milan Unit, Istituto di Ricerca Genetica e Biomedica (IRGB), CNR Milan Italy; ^2^ IRCCS Humanitas Research Hospital Milan Italy

**Keywords:** hematopoietic stem cells, MDS, miR‐127, miRNAs, self‐renewal

## Abstract

The hematopoietic system is sustained over time by a small pool of hematopoietic stem cells (HSCs). They reside at the apex of a complex hierarchy composed of cells with progressively more restricted lineage potential, regenerative capacity, and with different proliferation characteristics. Like other somatic stem cells, HSCs are endowed with long‐term self‐renewal and multipotent differentiation ability, to sustain the high turnover of mature cells such as erythrocytes or granulocytes, and to rapidly respond to acute peripheral stresses including bleeding, infections, or inflammation. Maintenance of both attributes over time, and of the proper balance between these opposite features, is crucial to ensure the homeostasis of the hematopoietic system. Micro‐RNAs (miRNAs) are short non‐coding RNAs that regulate gene expression posttranscriptionally upon binding to specific mRNA targets. In the past 10 years they have emerged as important players for preserving the HSC pool by acting on several biological mechanisms, such as maintenance of the quiescent state while preserving proliferation ability, prevention of apoptosis, premature differentiation, lineage skewing, excessive expansion, or retention within the BM niche. miRNA‐mediated posttranscriptional fine‐tuning of all these processes constitutes a safety mechanism to protect HSCs, by complementing the action of transcription factors and of other regulators and avoiding unwanted expansion or aplasia. The current knowledge of miRNAs function in different aspects of HSC biology, including consequences of aberrant miRNA expression, will be reviewed; yet unsolved issues will be discussed.

This article is categorized under:

RNA in Disease and Development > RNA in Disease
RNA in Disease and Development > RNA in Development

## Graphical abstract

Proper balance between self‐renewal, differentiation, and elimination of damaged cells ensures the maintenance of a constant pool of functional hematopoietic stem cells (HSCs). Examples of several miRNAs expressed in HSCs contributing to preserve this equilibrium are depicted.

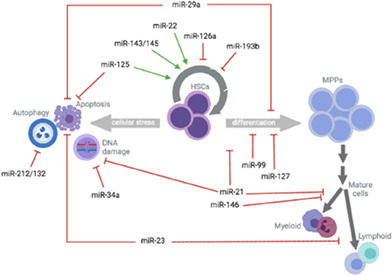

## 1 INTRODUCTION

Micro‐RNAs (miRNAs) are conserved 18–25 nucleotide‐long noncoding RNAs that regulate gene expression at the posttranscriptional level; they mainly destabilize target transcripts or repress their translation, upon binding to complementary 3′‐UTR regions of the target gene. The vast majority of the mRNA targets are recognized by the miRNA seed sequence, a highly conserved 6–8 nucleotide long sequence in the miRNA 5′‐region, but noncanonical recognition sites also exist (Seok et al., 2016). A single miRNA can bind to hundreds of target genes. On the other hand, several miRNAs can target a single mRNA or also a single pathway by binding to one or more mRNAs acting on that pathway (Qian et al., 2016). Their function is linked to almost every biological process including cell cycle progression, proliferation, differentiation, apoptosis, metabolism, response to environmental changes, and embryogenesis, to name some. Since their discovery (R. C. Lee et al., 1993), our knowledge on their biogenesis, function, and regulation has greatly deepened. Most miRNA genes are interspersed within the genome, although miRNA clusters, transcribed as a single polycistronic primary transcript and subjected to the same transcriptional regulation, are also frequent. miRNAs bearing the same seed sequence belong to the same family, although they are often located on different chromosomes. Canonical and noncanonical regulation of miRNA biogenesis and molecular functions have been recently reviewed elsewhere (O'Brien et al., 2018; Stavast & Erkeland, 2019; Treiber et al., 2019). Briefly, the primary transcript (pri‐miRNA) is transcribed by the RNAse pol II and initially processed by the microprocessor complex, composed by the RNAse III enzyme DROSHA and by the RNA‐binding protein DGCR8, to generate a precursor miRNA (pre‐miRNA) consisting of a 65 nucleotide‐long hairpin structure. The pre‐miRNA is exported into the cytoplasm and processed by the endonuclease DICER to produce the mature miRNA duplex. Individual miRNA strands bind to the RNA‐induced silencing complex (RISC) together with an argonaute (AGO) protein, which recruits factors to promote translational repression, mRNA deadenylation, and mRNA decay. However, several exceptions to these general rules have been recently discovered. For example, there are miRNAs located in the nucleus, where they are involved in the transcriptional activation or silencing of target genes, or in the modulation of alternative splicing; some miRNAs originated from short introns or from small nucleolar RNA transcripts do not need DROSHA processing; a DICER‐independent miRNA has also been described (Herrera‐Carrillo & Berkhout, 2017). DICER‐mediated processing of pre‐miRNAs can be controlled by different co‐factors and RNA‐binding proteins. A novel player, 5‐lipoxygenase (5‐LO), has been recently included among DICER modulators (Uebbing et al., 2021). 5‐LO is an enzyme involved in lipid mediator biosynthesis; its deletion decreases the expression of some of the miRNAs with key functions for hematopoietic stem cells (HSCs) (Uebbing et al., 2021).

In the present review, we will provide an overview of miRNA functions in the biology of HSCs, particularly of those involved in the maintenance of stemness properties. The different mature blood cells are produced in the bone marrow (BM) from HSCs via a series of intermediate progenitors and precursors. All steps along this hematopoietic hierarchy are subjected to miRNAs regulation; we will here focus exclusively on the cells at the apex of this hierarchy, the HSCs. HSCs are endowed with two fundamental characteristics: the ability to self‐renew throughout the entire lifespan of an individual, to guarantee a constant reservoir of blood cells, and at the same time, the capacity of multipotent differentiation, according to peripheral needs. Balancing these two properties is crucial to maintain the homeostasis of the hematopoietic system and of the entire organism. This equilibrium is achieved through cell‐intrinsic as well as cell‐extrinsic factors, including the interaction with cells belonging to the BM microenvironment, and virtually all biological mechanisms contributing to this balance are influenced by one or more miRNAs.

BOX 1 **Definitions of hematopoietic stem and progenitor cells**

- HSPC, Hematopoietic stem and progenitor cells: Heterogeneous hematopoietic cell population which includes a small proportion of stem cells, and mainly progenitors with different degrees of maturation and (multi)lineage potential. Human HSPCs are characterized by the expression of CD34 on their cell surface, while murine HSPCs are negative for lineage markers (Lin^−^) and co‐express cKit and Sca1 (LSK cells).
- HSC, Hematopoietic stem cells: They can be prospectively isolated to a relatively high purity using different combinations of cell surface markers in addition to CD34 (human) and to the LSK combination (mouse). Only in vivo functional assays can properly define them.

HSCs can be prospectively isolated both in human and mouse by fluorescence‐activated cell sorting using different combinations of cell surface markers (Laurenti & Göttgens, 2018). In the mouse, the cell population not expressing lineage‐associated markers (Lin^−^) and co‐expressing Sca1 and c‐Kit (LSK cells) contains mostly multipotent progenitor cells (MPPs) with different degrees of maturation, and a small proportion of bona fide HSCs, also called long‐term HSCs (Box 1). From a phenotypic point of view, adult murine HSCs are characterized by the absence of CD48 and of progenitor markers such as CD135 (also known as Flt3) and CD34, and by the expression of CD150. Other markers present in murine HSCs that can be used for their prospective isolation are CD105 (Pronk & Bryder, 2018) and/or EPCR. In human, hematopoietic stem and progenitor cells (HSPCs) are instead characterized by the expression of CD34; like mouse LSK, CD34^+^ cells include MPPs and a small proportion of HSCs, but also committed progenitors. CD34^+^ cells that are also Lin^−^, CD38^−^, CD45RA^−^, and CD90^+^ constitute one of the best enrichments for human bona fide HSCs, although other positive markers can be used such as EPCR and/or CD49f. Nevertheless, beyond their surface markers, HSCs must be defined functionally by their multipotent, long‐term repopulation capacity upon transplantation into syngeneic recipient mice, or as xenografts in immunodeficient murine hosts in case of human cells. We will here summarize and discuss the role of miRNAs in HSC biology.

## 2 miRNAS AND HSC STEMNESS

The hematopoietic system plays life‐essential roles thanks to its various blood elements: oxygen transport and delivery to all tissues of the body is performed by erythrocytes, wound healing by platelets, host defense and tumor surveillance by white blood cells. Most mature blood elements are short‐lived and must be promptly replenished to ensure proper functionality; thus, a pool of immature blood HSPCs must be constantly maintained, in the correct proportions between HSCs and MPPs. Hematopoiesis proceeds through well‐defined steps of differentiation, in a hierarchical organization with a small pool of multipotent HSCs at the apex. HSCs generate progenitors with higher proliferation capacity but progressively more restricted lineage potential, intermediate precursors, and finally mature cells. To constantly support the hematopoietic hierarchy, HSCs are endowed with self‐renewal as well as multipotent differentiation capacity throughout the life of the individual; balancing these two opposite fates is crucial to maintain the homeostasis of the hematopoietic system and avoid BM failure, immunodeficiency, onset of autoimmune diseases, or hematological disorders including malignancies. Different mechanisms must be tightly regulated to achieve this balance: to maintain the HSC pool, most HSCs are dormant or quiescent at any given time, with only a few in active state of proliferation, to be protected from DNA damage that can occur during cell cycle. At the same time, the capacity to exit the dormant state, proliferate, and differentiate according to peripheral requests without exhaustion must be preserved. All these mechanisms are primarily orchestrated by transcription factors and chromatin remodeling molecules in response to intrinsic or extrinsic signals, like cytokines, chemokines, or hormones, produced either by the surrounding microenvironment or by distant sites. However, posttranscriptional mechanisms, including the action of miRNAs, represent further checkpoints for fast and fine‐tuning the quantity of intracellular proteins essential for HSC function. The role of specific miRNAs in several differentiation steps along the hematopoietic hierarchy beyond the HSC stage has been reviewed elsewhere; we here focus on their role in maintaining long‐term HSC identity by affecting all mechanisms described above. In addition, miRNAs whose function has been described in HSPCs (murine LSK and/or human CD34^+^ cells) but their specific role in HSCs has not been dissected out, will not be considered here.

The majority of what is currently known on the role of miRNAs in HSCs is derived from mouse studies, where the expression of specific miRNAs has been altered with different methods (Table 1). Among the first evidences that miRNAs play a role in HSCs there is the observation that in the absence of Dicer, HSPCs display altered function and a higher rate of apoptosis (S. Guo et al., 2010). While the role of some miRNAs has been linked to HSC biology for several years now (miR‐125, miR‐126, miR‐29a, and miR‐193), the association of other miRNAs to HSC is quite recent (miR‐143/145, miR‐127‐3p, miR‐34a, and miR‐21) and will be integrated in the context of HSC maintenance mechanisms. A few examples of miRNAs associated to HSC‐related hematological disorders and involved in their pathogenesis will be also discussed.

**TABLE 1:** miRNAs expressed in HSCs

miRNA	Role in HSCs	Phenotype if DR^a^	Phenotype if OE^a^	KO phenotype^b^	TG phenotype^b^	Role in disease^c^	References
miR‐10a	Induces self‐renewal, drives lymphopoiesis	n.r.	Increase serial replating ability; upon TX: increase of LSK and of total BM cells	n.r.	n.r.	Present in MDS‐MSC MVs	Muntión et al. (2016); Wünsche et al. (2018)
miR‐21	Protects from radiation‐induced DNA damage	n.r.	n.r.	Increase of phenotypically defined HSCs, loss of HSC quiescence and long‐term reconstituting ability; myeloid versus lymphoid skewing	n.r.	Elevated in many types of cancer including myeloid leukemia	M. Hu et al. (2021); Panagal et al. (2018)
miR‐22	Induces cell cycle	n.r.	Increased serial‐replating ability	n.r.	Increased HSC self‐renewal and defective differentiation; MDS development	UR in MDS and leukemia; aberrant expression correlates with poor survival.	Song et al. (2013)
miR‐23a cluster	Lymphoid versus myeloid cell fate decision	n.r.	Increases myelopoiesis with reciprocal decrease in B lymphopoiesis	Increase in B lymphocytes and decrease in myeloid cells; increased BM cellularity	n.r.	n.r.	Ahn et al. (2013); Kurkewich et al. (2016)
miR‐23a/miR‐23b double KO	Lymphoid versus myeloid cell fate decision, protection from apoptosis	n.r.	n.r.	Decreased BM cellularity, increased lymphoid at the expense of myeloid development; decreased LSK, MPP, and HSC	n.r.	n.a.	Kurkewich et al. (2018)
miR‐29a	Limits HSC maturation, protects LSK from apoptosis	n.r.	Acquisition of self‐renewal capacity by myeloid progenitors, biased myeloid differentiation	Decreased HSPC numbers and clonogenic ability, decreased HSC self‐renewal, increased HSC cell cycling and apoptosis; HSCs with gene expression patterns similar to normal committed progenitors	n.r.	Expressed in AML	W. Hu et al. (2015)
miR‐34a	Protects from apoptosis and DNA damage	n.r.	n.r.	Increased HSC apoptosis and reduced repopulating ability	n.r.	Tumor suppressor	Zeng et al. (2019)
miR‐99	Inhibits HSC differentiation and cell cycle entry	Increased myeloid differentiation in vitro, reduced self‐renewal in vivo with increased LSK cycling and myelopoiesis	No phenotype	n.r.	n.r.	Expressed in AMKL and in AML LSC	Emmrich et al. (2014); Khalaj et al. (2017)
miR‐125a	Regulates clone size by inducing self‐renewal and preventing apoptosis	Impairs long‐term contribution to blood cell production	MPN‐like	MPD	n.r.	Increased in MDS	Emmrich et al. (2014); S. Guo et al. (2010); Luinenburg et al. (2021); Tatsumi et al. (2016); Wojtowicz et al. (2019); Wojtowicz et al. (2016); Wojtowicz et al. (2014)
miR‐125b	Protects from apoptosis, affects lymphoid versus myeloid differentiation	n.r.	MPD, enhanced hematopoietic engraftment upon xenotransplantation	n.r.	Eμ/miR‐125b‐TG mice: B‐cell malignancies	Involved in chromosomal translocations leading to B‐ALL, AML, and MDS	Bousquet et al. (2008); Emmrich et al. (2014); Enomoto et al. (2011); O'Connell et al. (2010)
miR‐125b‐2	Affects lymphoid versus myeloid differentiation	Impairs long‐term contribution to blood cell production	Lymphoproliferative disease	n.r.	n.r.		Ooi et al. (2010); Wojtowicz et al. (2014)
miR‐126a	Restrains HSC cell cycle entry	increases HSC proliferation without inducing exhaustion	impaired cell‐cycle entry, leading to progressively reduced hematopoietic contribution	n.r.	n.r.	Highly expressed in AML LSC; association of high miR‐126 with decreased survival of AML pts	Lechman et al. (2016); Lechman et al. (2012); Shen et al. (2008)
miR‐127‐3p	Limits HSC maturation while affecting proliferation	Loss of self‐renewal due to accelerated differentiation	Lymphopenia	n.r.	n.r.	UR in APML	Crisafulli et al. (2019)
miR‐212/132 cluster	Maintains HSC function with age acting on cell cycle and survival	n.r.	After TX: Initial HSC and mature cells expansion due to hyperproliferation, followed by a decline. HSC exhaustion; in vitro: no effect	HSC increase in aged mice, with decrease in LSK and mature cells; defect in HSC cycling in response to stress	n.r.	n.r.	Haetscher et al. (2015); Mehta et al. (2015)
miR‐142a‐3p	Controls HSC specification and T cell differentiation	By MO: reduced HSC numbers, loss of HSC master regulators, defective T cell development	n.r.	Decreased HSPC	n.r.	n.r.	X. Lu et al. (2013); X. Lu et al. (2015)
miR‐143/145	Required for HSC maintenance through suppression of TGFβ/DAB2 signaling	n.r.	n.r.	Reduced HSC number and activity; MPD in a proportion of aging mice	n.r.	DR in del(5q) MDS patient' HSPCs	Lam et al. (2018)
miR‐146a	Preserves HSC longevity and self‐renewal	n.r.	After TX: transient myeloid expansion, impaired BM reconstitution, reduced HSC survival	Loss of self‐renewal; transient increase of HSC and mature cells with age, followed by severe BM cell depletion in older mice; myeloproliferative disease or lymphoma with aging	Autoimmune lymphoproliferative syndrome upon constitutive, non‐targeted expression	DR in del(5q) MDS patient' HSPCs	Q. Guo et al. (2013); Mitsumura et al. (2018); Starczynowski et al. (2011); Zhao et al. (2013)
miR‐193b	Restricts HSC expansion by inhibiting cytokine signaling	n.r.	In vitro: lack of HSC expansion; after TX: lack of blood reconstitution	HSC expansion (phenotype and function) in mice older than 6mo; increase HSC cycling, shorter time for first division	n.r.	DR in AML	X. N. Gao et al. (2011); Haetscher et al. (2015)
miR‐320	Unknown	n.r.	n.r.	n.r.	n.r.	Prognostic in MDS	C. Wan et al. (2021)

Abbreviations: ALL, acute lymphoblastic leukemia; AMKL, acute megakaryoblastic leukemia; APML, acute promyelocytic leukemia; DR, downregulated; n.a., not applicable. nr, not reported; OE, over‐expressed; pt, patients; TX, transplant; UR, upregulated.

^a^ In BM, HSPCs or HSCs.

^b^ Related to hematopoiesis.

^c^ Hematopoietic diseases with HSPC origin.

### 2.1 Maintenance of HSC identity through cell‐intrinsic mechanisms

Most biological mechanisms that are in place to maintain HSC identity are a peculiarity of the adult hematopoietic system. In vertebrates, HSCs develop in the aorta–gonad–mesonephron (AGM) area of the dorsal aorta of day 10.5–11.5 or day 27–40 of mouse and human embryos, respectively (Dzierzak & Medvinsky, 2008; Julien et al., 2016). At later developmental stages they are found in the fetal liver, where they expand, and then finally migrate and lodge in the BM. The rate of proliferation and metabolic activity is different during the embryonal, fetal, and perinatal life compared with the adult, when HSCs are mostly quiescent. This review will mainly focus on the role of miRNAs in HSCs of adult individuals, for which more information is available, with only a brief reference to few miRNAs involved in HSC specification in Section 2.3.

#### 2.1.1 Prevention of cell cycle entry: miR‐126a, miR‐193b, and miR‐21

Different miRNAs contribute to prevent cell cycle entry and to restrain proliferation, thus avoiding HSC expansion when unnecessary. Unlike other cell types, in HSCs proliferation is a prerequisite for differentiation toward the subsequent steps in the hematopoietic hierarchy. In HSCs the fate of proliferation depends on the capacity to undertake symmetric and asymmetric cell divisions. With asymmetric cell division, one daughter cell remains HSC, while the other becomes a progenitor, thus the proportion between HSCs and differentiating cells is maintained. On the other hand, symmetric cell division can result in increased self‐renewal when both daughter cells are still HSCs, or to increased differentiation when both daughter cells become progenitors (Morrison & Kimble, 2006). Therefore, an excess of HSC proliferation can paradoxically result in two opposite outcomes: expansion (in some instances at the expenses of differentiation), or exhaustion and/or loss of functionality. For example, HSC expansion without exhaustion occurs when miR‐126a is experimentally downregulated. miR‐126a is differentially expressed in HSCs compared with lineage‐committed progenitors, but still highly expressed at the MPP stage. It prevents cell cycle entry by affecting PI3K/AKT signaling. Attenuation of its activity in mouse and human HSPCs results in expansion of phenotypically and functionally defined HSCs, without inducing exhaustion or malignancies, while overexpression prevents HSC cell cycle entry and reduces hematopoietic output in vivo (Lechman et al., 2012). Interestingly, as opposed to normal HSCs, prevention of cell‐cycle entry due to high miR‐126a expression is instead advantageous for leukemic stem cells (LSC), since their quiescent state confers them resistance to chemotherapy (Lechman et al., 2016).

Another example of miRNA whose function is linked to cell cycle is miR‐193b, identified as a regulatory feedback molecule restricting excessive HSC self‐renewal upon the activation of the TPO‐MPL‐STAT5 signaling. Expansion of HSCs in miR‐193b^−/−^ mice, as well as in primary and secondary recipients of miR‐193b^−/−^ cells, is not transient and does not ultimately result in HSC exhaustion, but rather in increased self‐renewal (Haetscher et al., 2015). This is justified not only by a higher proportion of HSCs that exit from their quiescent state, but also by a shorter time from first division at the single cell level. Moreover, an in vitro delay in differentiation was observed in HSCs from miR‐193b^−/−^ mice. miR‐193b is one of the miRNAs that we found to be expressed in HSCs and downregulated in the very first maturation step that from HSCs brings to Flt3^−^MPPs (Crisafulli et al., 2019) when loss of self‐renewal occurs.

Like miR‐126a and miR‐193b, the absence of miR‐21 causes HSC exit from quiescence and results in the expansion of phenotypically defined HSCs; nevertheless, these expanded HSCs are myeloid‐biased and less functional as demonstrated by the severely reduced reconstitution ability of BM cells from mice with miR‐21 conditional deletion. In detail, wild‐type (WT) recipients transplanted with BM cells from miR‐21‐conditional knock‐out (KO) mice died earlier than those transplanted with miR‐21‐positive BM cells in secondary non‐competitive BM transplantation (M. Hu et al., 2021). In competitive BM transplantation experiments, miR‐21 deficiency resulted in a lower proportion of donor‐derived cells in recipients' peripheral blood and LSK cells after primary and secondary transplants. miR‐21 role in maintaining HSC homeostasis is mediated by targeting Programmed cell death 4 (Pdcd4), thus supporting the nuclear factor kappa B (NF‐kB) pathway, already implicated in preserving HSC function (M. Hu et al., 2021). Thus, increased proliferation results in increased HSC self‐renewal when miR‐126 or miR‐193 are absent, or in HSC exhaustion without miR‐21.

#### 2.1.2 Induction of proliferation: miR‐125, miR‐143/145, and miR‐22

An excess of proliferation may be deleterious for HSCs, nevertheless the capacity to proliferate is a pre‐requisite to preserve both self‐renewal and differentiation. Overexpression of miR‐125a, mammalian ortholog of *lin‐4*, the first discovered miRNA in *Caenorhabditis elegans*, was shown to affect HSC expansion at the single cell level. This led to increased clone longevity, size, contribution to multilineage engraftment, and trafficking from the BM to the circulation after transplantation (Wojtowicz et al., 2019). Continuous miR‐125a expression appears to be necessary to induce HSC proliferation and self‐renewal (Luinenburg et al., 2021). Other miR‐125 family members (miR‐125b1 and miR‐125b2), which share with miR‐125a the same seed sequence, exert similar effects on HSCs when overexpressed through retroviral vectors (Wojtowicz et al., 2014). Contradictory data regarding the function of miR‐125b in lymphoid versus myeloid skewing have been reported (Ooi et al., 2010). However, properly regulated expression of miR‐125 in HSCs is crucial to maintain their correct overall pool size and blood cell numbers (S. Guo et al., 2010) thus reducing the likelihood of expansion of malignant clones. Indeed, miR‐125 overexpression in murine HSCs has been associated to myeloid skewing and to myeloproliferative neoplasm (MPN) development (S. Guo et al., 2012; Wojtowicz et al., 2014). Of note, also the absence of miR‐125 in KO mice results in myeloproliferative disorders (Tatsumi et al., 2016), and this is in line with the reported role for miR‐125 as tumor suppressor in vivo, both in mouse models and in patients (Berg et al., 2021). On the other hand, miR‐125a was found to be overexpressed in CD34^+^ cells from myelodysplastic syndrome (MDS) patients and to play a role in the disease (see below). The opposing roles for miR‐125a and for other family members described in the literature lead to the conclusion that fine tuning of miR‐125 expression must be achieved and regulated at any given time and differentiation step in the hematopoietic hierarchy. In this way, a correct balance of proliferation and quiescence, as well as of myeloid and lymphoid differentiation, prevents the development of hematopoietic malignancies. Given that the miR‐125a locus is highly conserved between human and mouse, the knowledge of how miR‐125a expression is controlled in HSCs would be of crucial interest.

Other miRNAs positively regulating HSC proliferation are miR‐143 and miR‐145, which are transcribed as a single primary transcript. In a mouse model with miR‐143/145 inactivation, depletion of HSCs was documented both in targeted mice and in mice transplanted with cells overexpressing the miR‐145 mRNA target DAB2, a positive regulator of TGFβ signaling, which dampens HSC proliferation. Decreased activity of miR‐143/145^−/−^ HSC was demonstrated by secondary colony assays (Lam et al., 2018). Since mice lacked both miRNAs, whether both exert this function or if one of the two is prominent it remains to be investigated.

Another example of miRNA inducing HSC proliferation is miR‐22, which emerged as being markedly upregulated in MDS patients (Song et al., 2013). Mice conditionally expressing miR‐22 in the hematopoietic compartment displayed increased HSC self‐renewal accompanied by myeloid skewing, ultimately leading to an MDS‐like disease. This is due to concomitant silencing of ten‐eleven‐translocation gene 2 (Tet2) and of phosphatase and tensin homolog (Pten).

#### 2.1.3 Restraining differentiation: miR‐29a, miR99, and miR‐127

Another crucial biological mechanism to preserve HSC maintenance is the inhibition of excessive differentiation, which may or may not be linked to increased HSC proliferation. Like miR‐126a, miR‐29a is differentially expressed in HSCs compared with lineage‐committed progenitors, but still highly expressed at the MPP stage. It limits HSC maturation in part through regulation of epigenetic changes by targeting DNA methyltransferase 3A (Dmnt3a) (W. Hu et al., 2015). Deletion of miR29a results in reduced number of both HSCs and MPPs, which display cell‐intrinsic self‐renewal defects in vivo. These defects are due to loss of quiescence and premature differentiation, with phenotypically defined HSCs from miR‐29a KO mice harboring a transcriptional profile resembling that of committed progenitors.

All three members of the miR‐99 family were found to be expressed at higher levels in mouse HSCs compared with more differentiated populations (Khalaj et al., 2017). miR‐99 inhibits both differentiation and cell cycle entry by targeting HOXA, thus maintaining HSC long‐term reconstitution ability. Functional inactivation of miR‐99 leads to HSPC depletion due to accelerated differentiation.

A further example of miRNA inhibiting differentiation is miR‐127, which is almost absent beyond the HSC developmental stage in steady state BM (Crisafulli et al., 2019). Inhibition of miR‐127‐3p function in HSPCs through a lentiviral‐sponge vector led to severe stem cell depletion, as assessed with serial transplantation assays. The observed HSC reduction was accounted for by their accelerated differentiation, in the absence of increased proliferation (Crisafulli et al., 2019), as opposed to what reported upon miR‐99 and miR‐29a inactivation. A peculiarity of miR‐127 is its strong HSC specificity; moreover, it emerged as the highest downregulated miRNA in HSCs from the Pbx1‐conditional KO mouse model, which are characterized by a profound self‐renewal defect and a tendency to differentiate prematurely (Ficara et al., 2008; Ficara et al., 2013). Despite its crucial function in maintaining HSC self‐renewal, miR‐127 is not expressed at high levels in HSCs; this is probably the reason why it has not been identified earlier among miRNAs regulating HSC biology. Even when purified with the best available combination of markers, phenotypically defined HSCs are still heterogeneous, and include cells with different degrees of differentiation potential and cell cycle state, as shown with experiments of single cell sequencing (Wilson et al., 2015). A selective expression of miR‐127 in a stem cell subpopulation might theoretically justify its apparently low abundance in the sorted HSCs.

#### 2.1.4 Protection from DNA damage: miR‐34a and miR‐21

Various mechanisms are in place to protect HSCs from different harms, including DNA damage that can occur physiologically during replication, or that can be induced by external insults such as radiation. One example of miRNA affecting this mechanism is miR‐34a, shown to promote irradiation‐induced DNA damage repair in HSCs. In the absence of miR‐34a, increased radiosensitivity in HSCs has been reported (Zeng et al., 2019), despite miR‐34a is not essential for steady‐state hematopoiesis. miR‐34a involvement in DNA damage repair is likely linked to promotion of homologous recombination (HR) and nonhomologous end‐joining (NHEJ) DNA repair processes. DNA damage in miR‐34a^−/−^ mice induces long‐term functional consequences: their HSCs displayed reduced quiescence up to at least 3 months after total body irradiation, and loss of long‐term reconstituting ability. DNA damage has been shown to induce aging of HSCs (Beerman et al., 2014; Flach et al., 2014), therefore miR‐34a activity might also be key to prevent premature aging (see also Section 2.2.1). A low but detectable level of expression of the mature form of miR‐34a in HSCs was observed also by our group (GEO Series accession number GSE113062; Crisafulli et al., 2019). However, only upon radiation or other DNA damage‐inducing agents, miR‐34a is likely induced as needed.

Another miRNA contributing to protect HSCs from radiation‐induced DNA damage (such as double‐strand breaks) is miR‐21, already mentioned in Section 2.1.1; mediators of this function might be the NF‐κB pathway, thrombopoietin‐mediated NHEJ in HSCs, or other not yet identified molecular pathways (M. Hu et al., 2021).

#### 2.1.5 Protection from apoptosis: miR‐23a/miR‐23b, and miR‐125

miR‐23a has been recently included among the HSC‐maintaining miRNAs due to its function in inhibiting apoptosis. miR‐23a gene codes for a miRNA cluster including miR‐23a itself, miR‐24‐2, and miR‐27a; a paralogous miRNA cluster gives rise to three mature miRNAs (miR‐23b, miR‐24‐1, and miR‐27b) with identical seed sequences to miR‐23a itself, miR‐24‐2 and miR‐27a, respectively. Deletion of miR‐23a alone did not result in HSPC defects, while miR‐23a^−/−^miR‐23b^−/−^ conditional double‐KO mice displayed decreased BM cellularity including fewer hematopoietic progenitors and HSCs, which also had a competitive disadvantage compared with WT or miR‐23a^−/−^ cells in a transplantation setting. Increased apoptosis was suggested as the mechanism at the basis of decreased HSPC populations (Kurkewich et al., 2018).

Decreased apoptosis had been also observed upon miR‐125a over‐expression (S. Guo et al., 2010). miR‐125b, which belong to the same family of miR‐125a as mentioned, is produced from a single miR‐99b/100‐125b polycistronic message. The combined activity of all three mature miRNAs converges to block the TGFβ pathway and amplify Wnt signaling thus escaping TGFβ‐induced apoptosis (Emmrich et al., 2014).

#### 2.1.6 Prevention of lineage skewing: miR‐23a/miR‐23b and miR‐21

To preserve multipotency, skewing to specific lineages must be prevented. In addition to affecting apoptosis as seen in Section 2.1.5, compound loss of miR23a/miR23b miRNAs gives rise to augmented CLP production at the expense of myeloid progenitors (Kurkewich et al., 2018). miR‐21 has the opposite effect compared with miR‐23a/miR‐23b, since its absence in HSCs leads to myeloid‐skewed differentiation at the expenses of the B‐lymphoid lineage (M. Hu et al., 2021). The described effects of miR‐21 deletion in HSCs underline how avoidance of excessive cell cycle, prevention of apoptosis, and preservation of multipotency are linked and all crucial to maintain HSC properties.

#### 2.1.7 Resistance to inflammatory stress: miR‐146a

Although miR‐146a is expressed in HSCs, it is more abundant along with maturation and it does not seem to be essential for hematopoietic development nor for steady‐state hematopoiesis; its function appear evident with aging or upon the occurrence of inflammatory stress. In general, miR‐146 expression level is modest, but it is upregulated by different inflammatory stimuli including LPS and proinflammatory cytokines, through NF‐kB direct regulation. It represses IRAK1 and TRAF6 through direct mRNA targeting, thus acting as feedback inhibitor for NF‐kB activation, since IRAK1 and TRAF6 are upstream of NF‐kB, and help resolving the inflammation‐induced immune reaction by reducing myelopoiesis.

#### 2.1.8 Other miRNAs maintaining self‐renewal

miR‐10a has recently been included among miRNAs positively affecting self‐renewal, although the biological mechanism through which miR‐10a exerts its function has not been clarified yet, nor its mRNA target(s). Importantly, it was found preferentially expressed in human cells with long‐term engraftment capabilities. Functionally defined miR‐10‐expressing HSCs were identified using gamma‐retroviral vector integration sites in gene therapy patients 6 years after autologous HSC transduction and transplantation (Wünsche et al., 2018). Overexpression of miR‐10 in murine HSCs resulted in increased replating ability in CFC assays and in augmented proportion of HSCs and MPPs upon transplantation, leading to increased BM cellularity and T cell lymphopoiesis.

The miR‐212 and miR‐132 cluster (or Mirc19) is enriched in HSCs and is upregulated during aging (Mehta et al., 2015). Mirc19 regulates HSC cycling, function, and survival through autophagy thus balancing over time HSC maintenance and normal immune cell production.

### 2.2 Maintenance of HSC identity through the microenvironment

In post‐natal life, hematopoiesis occurs mainly within the BM, where HSCs are in close contact with cells belonging to the microenvironment. Niche cells include osteoblast bone‐lining cells, mesenchymal stromal cells (MSCs), macrophages, megakaryocytes (MKs) and endothelial cells. In one view of the BM microenvironment, “dormant” HSCs are located in the endosteum in association with arterioles, while “active” HSCs reside in vascular niches and are located adjacent to sinusoids. High resolution image studies revealed that indeed HSCs reside mainly close to sinusoids, to Cxcl12^+^ stromal cells, and to MKs (Kokkaliaris et al., 2020), although the picture is evolving continuously. Cells belonging to the BM microenvironment provide HSCs with a tridimensional niche that nurtures them and maintains their stem cell characteristics by releasing growth factors and/or other molecules, by inducing intracellular signals through cell–cell interaction, and by retaining them in the BM or releasing them when needed. miRNAs are emerging as additional players in this complex network, either by affecting the function of niche cells, or by being released and up taken by HSCs.

#### 2.2.1 Vesicles produced by cells belonging to the HSC niche

Extracellular vesicles (EVs) are membranous nanoparticles (30–10,000 nm) secreted by all cell types, carrying bioactive proteins, lipids and nucleic acids including miRNAs. They can be transferred to other cells to mediate both local and distant intercellular communication (Valadi et al., 2007). EVs include microvesicles (MVs, 50–1000 nm), generated by calcium mediated budding and cleavage of the plasma‐membrane and remodeling of the cortical cytoskeleton, as well as exosomes (30–150 nm), which mature from endosomes and then fuse to the inner layer of the plasma membrane for release (Butler et al., 2018). MSCs produce both MVs and exosomes. BM‐MSC‐derived EVs were shown to contain several miRNAs and to alter gene expression profile of cord‐blood CD34^+^ cells exposed to them ex‐vivo (De Luca et al., 2016). Among BM‐MSC‐EV contained miRNAs, miR‐21, and miR‐22 are known to play a role in HSCs (Tables 1 and 2); some of their predicted target mRNAs were indeed downregulated in CD34^+^ cells upon exposure to EVs (De Luca et al., 2016).

**TABLE 2:** HSC related miRNAs expressed in other adult tissue‐specific stem cells

miRNA	Stem cell	Function	References
miR‐21	MSC^a^	Promotes osteoblast, osteocyte, and adipocyte differentiation; limits cartilage differentiation	Sekar et al. (2015); Vail et al. (2021)
	Periodontal ligament stem cells (PDLSCs)	Promotes osteogenic differentiation following orthodontic force	H. Huang et al. (2018)
miR‐22	BM‐MSC^a^	Inhibits osteoblast differentiation	Yin et al. (2020)
	PDLSCs	Promotes osteoblast differentiation	Yan et al. (2017)
	Adipose tissue derived MSC (ADMSCs)	Inhibits adipogenesis, promotes osteogenesis	S. Huang et al. (2012)
	Fibro/adipogenic progenitors (FAPs)	Prevents adipogenesis	Lin et al. (2020)
	Hair follicle stem cells (HFSCs)	Inhibits proliferation and differentiation, regulates hair cycle	Cai et al. (2020); Yuan et al. (2015)
miR‐23	BM‐MSC	Suppresses osteogenic differentiation	K. Jiang et al. (2020)
	Myoblasts	Induces skeletal muscle differentiation	Mercatelli et al. (2017)
miR‐29a	BM‐MSC	Promotes osteogenic differentiation, inhibits cartilage formation, inhibits proliferation, prevents senescence	Guérit et al. (2014); Jung et al. (2020); Kapinas et al. (2010); Tan et al. (2018); Y. Zhang and Zhou (2015)
	Multipotent adipose stem cells (MADS)	Inhibits adipogenesis	Glantschnig et al. (2019)
	Myoblasts	Improves differentiation into myotubes	X. H. Wang et al. (2011)
	Neural stem/progenitor cells (NSPCs)	Promotes neural differentiation, neurite outgrowth and complexity, still maintaining stemness	Y. Gao et al. (2020); Ma et al. (2020)
miR‐34a	BM‐MSC	Inhibits/promotes osteogenic differentiation; proapoptotic and prosenescence roles	Chen et al. (2014); Liu et al. (2019); Pi et al. (2021); F. Zhang et al. (2015)
	Adipose‐derived stem cells (ADSCs)	Promotes senescence rather than differentiation	Park et al. (2015)
	Dental pulp stem cells (DPSC)	Induces senescence	S. Zhang et al. (2021)
	Neural stem cells (NSCs)	Induces/inhibits differentiation	Aranha et al. (2011); Morgado et al. (2015)
miR‐125	BM‐MSC	Inhibits osteoblast differentiation	Tu et al. (2016)
	Glial progenitor cell (GPC)	Primes astrogliogenesis	Shenoy et al. (2015)
miR‐126a	BM‐MSC	Promotes proliferation and endothelial differentiation while inhibits apoptosis and osteogenic differentiation	Kong et al. (2020)
	DPSC	Induces apoptosis	Ge et al. (2021)
	Endothelial progenitor cells (EPCs)	Maintains stemness	Pei et al. (2020)
miR‐127‐3p	Myoblasts	Inhibits proliferation, enhances differentiation	Li et al. (2020); Zhai et al. (2017)
miR‐212/132	BM‐MSC	Suppresses osteogenic and promotes chondrogenic differentiation	Y. Zhang, Jiang, et al. (2020); Zhou et al. (2018)
	PDLSCs	Inhibits osteogenic differentiation	Xu et al. (2019)
	Precartilaginous stem cells (PCSCs)	Promotes proliferation and inhibits apoptosis	F. Y. Zhang, Zhen, et al. (2020)
	NSCs	Induces hippocampal neurogenesis	Walgrave et al. (2021)
miR‐143/145	BM‐MSC	Negative regulator of osteogenesis and of EC differentiation	Cha et al. (2016); Feng et al. (2018)
	ADSCs	Negative regulator of osteogenesis	Hao et al. (2018)
miR‐146a	BM‐MSC	Inhibits proliferation, promotes immune‐modulatory properties, and osteoblast differentiation/suppresses osteoblastogenesis and bone formation	Cui et al. (2020); Kuang et al. (2017); Zheng et al. (2021); Zhou et al. (2016)
	ADSCs	Inhibits osteogenic differentiation	S. Wan et al. (2020)
miR‐193b	BM‐MSC	Promotes chondrogenesis/inhibits early chondrogenesis, promotes proliferation	Hou et al. (2015); Meng et al. (2018); J. Wang et al. (2012)
	ADSCs	Promotes adipogenesis	Mazzu et al. (2017)

^a^ MSC is the acronym of “mesenchymal stromal cells” in some studies, or of “mesenchymal stem cells” in others. MSCs are multipotent clonogenic progenitors that give rise to several cell types including osteoblasts, osteocytes, cartilage, and adipocytes. The definition of MSC as “stem cells” is sometimes controversial, mainly because it is not clear if their multipotency is exerted in vivo at the single cell level. All studies mentioned in Table 2 consider MSC as stem cells. MSC can be isolated from different tissues; BM‐MSC are BM‐derived.

The role of miRNA‐containing exosomes secreted by MSCs has been also associated to aging. In detail, MSC exosomes from young mice contribute to rejuvenate HSCs upon ex‐vivo exposure; they were shown to contain significant lower levels of miR‐34a compared with exosomes from aged MSCs (Kulkarni et al., 2018). The role of miR‐34a contained in MSC‐secreted exosomes has been linked to its ability to target the autophagy‐related protein Sirtuin, thus reducing autophagy. This in turn accelerates aging, since autophagy protects HSCs from metabolic stress (Ho et al., 2017). Fine tuning of miR‐34a is likely crucial: in steady state hematopoiesis under physiological conditions miR‐34a levels must be kept low both in HSCs (see Section 2.1.4) and in MSCs serving as HSC niche.

As mentioned previously and reviewed elsewhere (Ghosh et al., 2021), MKs are also part of the HSC niche. In the BM, MKs localize in close proximity to HSCs, promoting their quiescence and contributing to their retention within the BM. MVs produced by MKs have been shown to be internalized by HSPCs, to contain miRNAs, and to induce differentiation into MKs (J. Jiang et al., 2017). Whether miRNAs contained in MK MVs also participate to the maintenance of HSC quiescence it remains to be investigated.

#### 2.2.2 HSC‐supporting ability by niche cells: miR‐23a

The chemokine CXCL12 regulates the interaction between HSCs and BM stromal cells (BMSC). An intact CXCL12‐CXCR4 signaling is crucial to maintain the quiescent HSC pool and its retention within the BM (Sugiyama et al., 2006). CXCL12 produced specifically by BM MSC (but not by other cells belonging to the BM microenvironment) has been shown to be essential for HSC self‐renewal and reconstitution ability (Agarwal et al., 2019). However, temporary HSC release from the BM is physiologically needed in response to peripheral stimuli and must be properly regulated at any given time. miR‐23a (see also Section 2.1.6) was shown to be expressed in primary BMSC, to be upregulated upon TGFβ1 exposure, and to inhibit CLCX12 production in BMSC by direct binding to its 3′‐UTR (Arabanian et al., 2014). Additional functional studies are needed to further clarify the role of stromal miR‐23a in vivo; however, it is tempting to speculate that a TGFβ1‐mediated mechanism may be responsible for miR‐23a‐mediated reduction of CXCL12, leading to HSC release from the BM.

## 3 MIRNAS AND CONTROL OF HSC DEVELOPMENT

During embryonic development hematopoiesis occurs in two waves, primitive and definitive. The first blood cells appear in the yolk sac and in placenta and include primitive red blood cells and macrophages that do not arise from HSCs. Except for some tissue‐resident macrophages and for a portion of other macrophage‐related cells such as microglial cells and osteoclasts, most of these primitive blood cells do not persist until later stages of embryonic and fetal development nor in the adult. As mentioned earlier, definitive hematopoiesis starts mainly in the AGM, where HSCs and progenitors, from which all other blood cells will derive, are generated from the hemogenic endothelium. Only a few miRNAs have been described to play a role in this process.

### 3.1 Endothelial‐to‐hematopoietic transition

Endothelial‐to‐hematopoietic transition (EHT) is the mechanism through which definitive HSPCs develop from hemogenic endothelial cells (hemECs) during embryogenesis within AGM. Regulation of this process by miR‐223, a miRNA whose function in the adult is mainly linked to progenitors and mature blood cells rather than HSCs, has been recently demonstrated using a zebrafish model (Kasper et al., 2020). miR‐223 limits hemEC and HSPC production since in its absence proliferation and delamination in the AGM are increased, with consequent excessive expansion of lymphoid and myeloid progenitors and of differentiated cells in secondary hematopoietic organs. In hemECs miR‐223 regulates N‐glycan biosynthesis by binding to the 3′‐UTR of genes encoding enzymes involved in N‐glycosylation, thus affecting glycan profiles of proteins acting on EHT (Kasper et al., 2020).

Similarly to miR‐223, the function of miR‐142 in the adult hematopoietic system is mainly linked to mature cells, while during development miR‐142a‐3p has been shown to control HSC specification and differentiation both in zebrafish and in mouse AGM (X. Lu et al., 2013). In detail, morpholino‐mediated miR‐142a‐3p knock‐down affected specification of the hemogenic endothelium, which in turn attenuated the emergence of the earliest HSCs in the embryos. miR‐142a‐3p targets Irf7, which acts as a negative regulator of HSC development and differentiation into the T‐cell lineages.

## 4 miRNAs, HSCs, AND HEMATOLOGICAL DISORDERS

miRNAs aberrant expression has been documented in hematological and non‐hematological cancers, where they can act either as oncomirs or as tumor suppressors. Description of the role of miRNAs in hematological malignancies is beyond the scope of the present review. However, few examples of miRNAs whose function in normal HSCs has been mentioned here will be addressed, referred to MDS and to chronic myelomonocytic leukemia (CMML), as examples of hematological disorders with a clear HSC origin.

### 4.1 Myelodysplastic syndromes

MDS are clonal hematological disorders characterized by dysplasia and inefficient hematopoiesis leading to peripheral cytopenia, with high risk of transformation to acute myeloid leukemia (AML) (Sperling et al., 2017). HSCs have been demonstrated to be the disease‐initiating cells in MDS (Mortera‐Blanco et al., 2017; Pang et al., 2013; Woll et al., 2014). A consensus on different miRNA profiles of MDS patients versus healthy controls or in patients who underwent leukemic transformation has not been reached. This is mainly due to the different techniques used for miRNA isolation and analysis, and to the different cell sources, which include BM mononuclear cells, total BM aspirate, BM MSC, total blood plasma, with only few studies having used CD34^+^ HSPCs, as recently reviewed (Bauer et al., 2020). Therefore, in addition to the technical differences in the various studies, most authors have profiled very heterogeneous samples, and it is hard to extrapolate which miRNA plays a role in MDS stem cells, also considering that no data have been reported on purified HSCs from MDS patients.

#### 4.1.1 MDS‐associated miRNAs

The most frequent chromosomal aberration in MDS is the deletion of chromosome 5q, called del(5q) (Lam et al., 2018). The commonly deleted region (CDR) in del(5q) MDS includes miR‐143 and miR‐145, which are expressed at significantly lower levels in CD34^+^ cells from del(5q) MDS patients. Loss of miR‐143/145 reduces both normal and MDS HSC activity by inducing TGFβ signaling. The CDR in del(5q) MDS also encompasses another HSC‐related miRNA, miR‐146a.

Other recurrent chromosomal translocations in MDS affect the locus of miR‐125b resulting in its overexpression (Bousquet et al., 2008). In MDS patients, also the other miR‐125 family member, miR‐125a, was found to be overexpressed and to negatively correlate with survival (Gañán‐Gómez et al., 2014). It has been suggested that this miRNA could serve as prognostic marker and as potential therapeutic target in MDS, although differences exist in the various MDS subgroups, since the same miRNA was found downregulated in high‐risk MDS (Solly et al., 2017).

Additional recently identified potential prognostic biomarkers in MDS are miR‐320 family members (C. Wan et al., 2021), with miR‐320c and miR‐320d being associated with high numbers of BM blasts. Although the observation of this association has not been followed by functional studies yet, correlation with blasts is in accordance with the high expression of miR‐320 in highly purified HSCs and their immediate progeny of Flk2^−^MPPs that we have documented in murine BM (GEO Series accession number GSE113062; Crisafulli et al., 2019).

Mutations in genes involved in the spliceosome, thus also in pre‐miRNA splicing, are frequent in MDS (SF3B1, SRSF2, U2AF1, and ZRSR2; Bersanelli et al., 2021). In patients harboring one of these mutations, alteration of the expression of one or more HSC‐associated miRNAs has been recorded (miR‐21, miR‐22, miR‐125a, miR‐143/145), as summarized elsewhere (Bauer et al., 2020). In MDS miR‐21 mediates hematopoietic suppression by targeting Smad7, a negative regulator of the TGFβ pathway (Bhagat et al., 2013), while miR‐22 upregulation correlates with poor survival (Song et al., 2013).

Lastly, MVs from MDS patients have been shown to contain miR‐10, a miRNA involved in the maintenance of HSC self‐renewal (Section 2.1.9), to be able to be incorporated in CD34^+^ cells in vitro, and to affect their viability and clonogenic ability (Muntión et al., 2016).

### 4.2 Chronic myelomonocytic leukemia

Like MDS, CMML arises from HSCs. As in other cancers, in CMML tumor‐suppressor genes, including those coding for miRNAs, are often silenced through hypermethylation of their promoter regions. Accordingly, hypomethylating agents (HMAs) represent the current therapeutic approach. Little is known about HMA targets; however, the upstream region of miR‐125a has been recently identified as one of them (Berg et al., 2021) and this might be partially responsible for the anti‐leukemic effect of these drugs. miR‐125a downregulation has been documented in CMML (Berg et al., 2021) and in high‐risk MDS (Solly et al., 2017), as previously mentioned. Preliminary data suggest that patients with clinical response to HMAs are the ones showing the highest miR‐125 upregulation after treatment. Therefore, it would be interesting to test if the level of HMA‐induced miR‐125 upregulation in vitro might serve as a prognostic readout to prospectively identify HMA responders and thus apply the most suitable treatment.

## 5 miRNAs AND OTHER TISSUE‐SPECIFIC ADULT STEM CELLS

Several of the miRNAs whose function is reviewed above play critical roles also in tissue‐specific adult stem or progenitor cells other than HSCs; examples are provided in Table 2. Only for some of them the function is similar to the one described for HSCs. For instance, while miR‐22 induces proliferation and inhibits differentiation in HSCs, in hair follicle stem cells it prevents cell cycle. Moreover, it can have a positive or negative effect on differentiation depending on the stem cell type. Likewise, in MSCs miR‐21 has been implicated in regulating differentiation; however, a specific role in preventing cell cycle entry or DNA damage, as described in HSCs, has not been studied in detail in these cells. Other examples are miR‐34a, miR‐126, and miR‐127. In MSCs miR‐34a exerts a pro‐apoptotic function rather than a protective role as in HSCs; in addition, it is involved in differentiation of various stem cell types, although opposite functions have been reported (Table 2). miR‐126a has proproliferative and prodifferentiation roles in MSCs rather than a function in restraining cell cycle entry. In myoblasts miR‐127 induces differentiation rather than preventing it as in HSCs. A consensus has not been reached on the role of miR‐146 and miR‐193 in MSCs (Table 2). On the other hand, like what described in HSCs, in different stem cell types miR‐23 and miR‐125 are involved in cell fate decision. In MSCs miR‐29a prevents maturation toward cartilage and adipocytes, however it induces osteoblastic differentiation; a positive role in differentiation has been reported in myoblasts and in neural stem/progenitor cells.

In general, the effect of each miRNA appears to be cell and context specific.

## 6 miRNAs, HSCs, AND THERAPY

miRNA mimics and miRNA inhibitors are promising novel therapeutic agents (T. X. Lu & Rothenberg, 2018). Regarding the hematopoietic system, potential applications have been envisioned both in regenerative medicine and as anticancer drugs; few examples will be quoted here.

### 6.1 miRNA‐based anticancer drugs

Strategies for miRNA‐based anticancer treatments consist in miRNA inhibition, to reduce expression of oncomiRs, or miRNA replacement, to increase expression of a miRNA acting as tumor suppressor. miRNA functional inhibition can be achieved using antagomiRs, antisense oligonucleotides containing complementary sequences of endogenous miRNAs, or through miRNA sponges, consisting of plasmid constructs containing several target sequences to sequester specific miRNAs. miRNA overexpression is mediated by transfection of double‐stranded miRNA mimics, or through viral vectors. Some of these approaches have highlighted the difficulty of delivering naked miRNAs (negative charge, undesired off‐target effects, and short miRNA half‐life in vivo). New methods employing nanoparticles are offering better outcomes as shown in several preclinical studies reviewed elsewhere (S. W. L. Lee et al., 2019). Phase 1 and 2 clinical trials are ongoing to modulate the expression of different miRNAs for the treatment of various type of cancer or of other diseases; further research is needed to ascertain if these approaches will translate in clinical practice for hematological malignancies in which the cell of origin is a mutated HSC.

### 6.2 miRNAs as therapeutics in regenerative medicine

Temporary ex‐vivo miR‐125a overexpression has been proposed to exploit the beneficial effects of miR‐125 in enhancing HSC self‐renewal without the risk of inducing leukemia, with the aim of expanding HSCs for therapeutic purposes such as prior to transplant (Luinenburg et al., 2021). This strategy would be particularly useful when low HSC numbers are available such as in cord blood units (Wojtowicz et al., 2016).

The specific expression of some miRNAs in HSPCs as compared with their differentiated progeny has been exploited for improving the selectivity of lentiviral vectors for gene therapy purposes. To this aim, miRNA target sequences were inserted within the vector (to function as sponges) for selectively silencing the transgene in HSCs. This strategy would consent the protection of HSCs from potential toxicity of the transgene, while allowing its proper expression in more mature cells where it is required for therapy (Gentner et al., 2010).

## 7 CONCLUSION

During the past 10 years, prominent roles for miRNAs in several aspects of hematology have emerged, including specific functions in HSC biology. A plethora of miRNAs are indeed involved in maintaining the defining HSC characteristics of long‐term self‐renewal and multipotent differentiation ability. They represent further, post‐transcriptional checkpoints to fine‐tune regulation of specific genes whose expression is triggered by the concert action of transcription factors and chromatin modifiers. This additional level of regulation represents an important safety mechanism to ensure the homeostasis of the hematopoietic system starting from preservation of the stem cell pool. miRNAs take part in the regulation of virtually all biological processes that contribute to maintain stemness, such as temporary induction or inhibition of proliferation according to body needs, protection from apoptosis or DNA damage, preservation of multipotent differentiation capacity by preventing lineage skewing or premature differentiation, interaction with the microenvironment to ensure retainment within the BM, and resistance to inflammation‐induced damage.

Despite all the advancements in the knowledge of miRNA roles in HSCs, several aspects still need to be explored. First, one important mechanism to preserve the HSC pool is the choice between asymmetrical and symmetrical cell division, and in the latter case between self‐renewing proliferation versus generation of two differentiating cells; to our knowledge, no miRNA has been associated to this process yet. Second, the mRNA target(s) of some of the miRNAs whose function has been linked to HSCs are not known, or only a few of the putative targets for each miRNA have been investigated. Third, the rarity of HSCs in the BM has hampered thus far the possibility to perform deep proteomics studies to identify mRNA targets when the miRNA‐mRNA interaction results in reduction of protein levels rather than mRNA degradation. Last, contradictory roles have been reported for some of the described miRNAs. One example is miR‐125, defined either as an oncomir or as a tumor suppressor both in solid tumors and in hematological malignancies, even those with a stem cell origin. Different experimental approaches among distinct laboratories, technical aspects linked to differences in prospective isolation of HSCs rather than HSPCs, constitutive versus conditional KO and/or transgenic animal models, data obtained by gene deletion as opposed to ectopic expression, might all partially contribute to explain the described disparities. Nevertheless, the correct amount of each miRNA at any given time, developmental stage, cell cycle phase and cellular context is likely crucial, and both excess or reduction are deleterious and might paradoxically bring to similar outcomes. Which are all the molecular regulators of miRNAs expression level in HSCs, and especially how the rapid downregulation beyond the HSC stage is achieved, are still open questions for most of HSC‐associated miRNAs and require further investigation. Solving these issues will help dissecting the role of miRNAs in hematological disorders and designing novel miRNA‐based therapeutic strategies.

We apologize to those investigators whose work we were unable to cite for space limitations.

## CONFLICT OF INTEREST

The author declares no conflict of interest.

## AUTHOR CONTRIBUTIONS

**Laura Crisafulli:** Visualization (supporting); writing – original draft (supporting); writing – review and editing (equal). **Francesca Ficara:** Conceptualization (lead); supervision (lead); visualization (lead); writing – original draft (lead); writing – review and editing (equal).

## RELATED WIREs ARTICLES

MicroRNAs as therapeutic targets in human cancers

Mechanisms controlling hematopoietic stem cell functions during normal hematopoiesis and hematological malignancies

## Data Availability

Data sharing is not applicable to this article as no new data were created or analyzed in this study.
